# Investigation of antioxidant, antibacterial, antidiabetic, and cytotoxicity potential of silver nanoparticles synthesized using the outer peel extract of *Ananas comosus* (L.)

**DOI:** 10.1371/journal.pone.0220950

**Published:** 2019-08-12

**Authors:** Gitishree Das, Jayanta Kumar Patra, Trishna Debnath, Abuzar Ansari, Han-Seung Shin

**Affiliations:** 1 Research Institute of Biotechnology & Medical Converged Science, Dongguk University-Seoul, Gyeonggi-do, Republic of Korea; 2 Department of Food Science and Biotechnology, Dongguk University‐Seoul, Gyeonggi‐do, Korea; 3 Department of Obstetrics and Gynecology, Ewha Medical Research Institute, Ewha Womans University Medical School, Seoul, Republic of Korea; VIT University, INDIA

## Abstract

Currently, green nanotechnology-based approaches using waste materials from food have been accepted as an environmentally friendly and cost-effective approach with various biomedical applications. In the current study, AgNPs were synthesized using the outer peel extract of the fruit *Ananas comosus* (AC), which is a food waste material. Characterization was done using UV–visible spectroscopy, X-ray diffraction (XRD), Fourier transform infrared (FT-IR) spectroscopy, scanning electronic microscopy (SEM) and energy-dispersive X-ray spectroscopy (EDX) analyses. The formation of AgNPs has confirmed through UV–visible spectroscopy (at 485 nm) by the change of color owing to surface Plasmon resonance. Based on the XRD pattern, the crystalline property of AgNPs was established. The functional group existing in AC outer peel extract accountable for the reduction of Ag^+^ ion and the stabilization of AC-AgNPs was investigated through FT-IR. The morphological structures and elemental composition was determined by SEM and EDX analysis. With the growing application of AgNPs in biomedical perspectives, the biosynthesized AC-AgNPs were evaluated for their antioxidative, antidiabetic, and cytotoxic potential against HepG_2_ cells along with their antibacterial potential. The results showed that AC-AgNPs are extremely effective with high antidiabetic potential at a very low concentration as well as it exhibited higher cytotoxic activity against the HepG_2_ cancer cells in a dose-dependent manner. It also exhibited potential antioxidant activity and moderate antibacterial activity against the four tested foodborne pathogenic bacteria. Overall, the results highlight the effectiveness and potential applications of AC-AgNPs in biomedical fields such as in the treatment of acute illnesses as well as in drug formulation for treating various diseases such as cancer and diabetes. Further, it has applications in wound dressing or in treating bacterial related diseases.

## Introduction

Nanotechnology is a modern exploration field that involves design, synthesis, and employment of particles ranging in size from around one to hundred nanometers [1]. A number of physical and chemical methods were established and were widely used for synthesizing silver nanoparticles, considering the application of nanoparticles in biomedical fields [1, 2]. The use of silver nanoparticles comprising groups of silver atoms has gained popularity owing to their distinct properties [3]. To obtain essential products and also to further reduce or eradicate the waste materials produced, green synthesis of the nanoparticle is currently an emphasized area of investigation. Further investigation regarding the application of feasible silver nanoparticle-based approaches is necessary [4]. Nanotechnology is a developing research area encompassing the fields of biology, medicine, and engineering. In this regard, nano-biotechnologyis a unique multidisciplinary field which facilitates the use of nanoparticles in biomedical settings via green approaches [5].

Among nanoparticles made using metals such as Au, Ag, Ce, Pt, Pd, and Zn [6], AgNPs are well known for their beneficial effects owing to their antidiabetic, anti-oxidative, antibacterial, and cytotoxic activities [4, 7, 8]. It is also used as an antiseptic agent for long times [9]. Because of their broad-spectrum antimicrobial potential [10], AgNPs have been widely integrated into numerous products such as household antiseptic sprays, wound dressing agents, and antimicrobial coating agents for medical devices used to sterilize air, textiles, and surfaces [11]. For AgNPs, the biological synthetic method is beneficial over physicochemical techniques as it is cost-effective, environment-friendly, and easy for mass production [12]. The biologically synthesized silver nanoparticle is biocompatible, and it can be safely used for various therapeutic applications.

Using agricultural fruit peel waste in the synthesis of AgNPs is an ecofriendly opportunity [9]. *Ananas comosus* (L.) Merr. is the most popular edible member of the family *Bromeliaceae*. It is one of the leading marketable fruit crops worldwide, which is grown in numerous tropical and subtropical nations. In various native cultures, it has been used as medicine [13]. Crude extract from pineapple has various medicinal qualities [14]. AC fruit is normally used as food, and its outer peel is a waste byproduct; thus, using the peels in pharmaceutical applications proves to be economical. AC peels are sources of nutrients and bioactive compounds like proteins, minerals, lipids, vitamin C, phenolic compounds, flavonoids, carotenoid, etc. [14–16]. These bioactive compounds present in AC peel have the potential to reduce metal ions and stabilize the synthesized nanoparticles all through their growth [9].

The predominant polyphenolic compounds present in AC peels were gallic acid, ferulic acid, chlorogenic acid, catechin, and epicatechin [17, 18]. These polyphenolic compounds exhibit their antioxidant potential [19]. Methanolic extract of AC fruit peel also shows anti-rheumatic activity [20]. It has been reported that AC peel extract has antioxidative and antibacterial potential, which can be useful in food applications [9, 21, 22]. The extracts of AC peels or extracts of the fermented fruit can be used as a novel candidate in therapeutic approaches for cancer [23]. AC leaf extract possesses substantial antidiabetic activity, which in turn is partially because of its antioxidative nature [24]. Previous research revealed that AC fruit residue can be potentially utilized as a nutraceutical against diabetes and related problems [25]. Consequently, peels of AC fruits can be used as novel constituents in food preparations that are beneficial to health [14].

Earlier, very few studies have been conducted on the synthesis of AgNPs using the AC fruit outer peel extract and evaluation of its antimicrobial and antioxidant activity. However previous studies have used only few parameters to investigate the bio-potential of the said AgNPs. Hence, the objective of the current study was to synthesize AgNPs using the outer peel extracts of AC by employing a green-synthesis methodology and its characterization using UV-Visible spectroscopy, SEM, EDX, XRD, FTIR, analyses technique followed by investigation of its bio-potential using a number of assays and activities such as antidiabetic, antioxidative, antibacterial, and cytotoxic potentials.

## Materials and methods

### Materials and preparation of the peel extract

*Ananas comosus* fruit -was purchased from Goyangsi market, Republic of Korea. All chemicals used in the study were of analytical grade and were purchased from authorized firms. The outer peel of AC fruit (which is basically a waste product) was collected and cut into small pieces and perfectly dried using a tissue paper. Dry small pieces of the peels (weighing a total of 120 g) were immersed in 550 mL of deionized water in a 1000 mL Erlenmeyer flask and boiled for 20–30 min under constant stirring conditions. After boiling, when the mixture is cooled to room temperature, it was filtered using Whatman No. 1 filter paper, and the filtrate (AC) was collected in a bottle and stored at 4°C until further use.

### Synthesis of AgNPs using outer peel extracts of AC

AgNPs were biosynthesized under normal laboratory condition. Concisely, three sets of 100 mL of aqueous 1 mM AgNO_3_ (Sigma-Aldrich, St. Louis, Missouri, USA) solution present in a 250-mL conical flask were gradually added on a drop-by-drop basis to 10 mL of AC extracts under continuous stirring conditions at room temperature [26, 27]. From the reaction mixture of all the three sets, the biosynthesis of AgNPs was observed visually on the basis of a change in the color of the reaction mixture at regular time intermissions. After incubation for 24 h, the reaction mixture, which comprised the biosynthesized AgNPs, was centrifuged at 10,000 rpm for 30 min using a high-speed centrifuge. The supernatant was thrown away, and the pellet was washed three to four times with double distilled water and again centrifuged to remove any unwanted and unattached AC extracts. Subsequently, the pellets were dried at 55°C and in an airtight vial for further investigation.

### Characterization of biosynthesized AgNPs

The biosynthesized AgNPs were morphologically, physically, and chemically characterized using diverse analytical techniques such as UV-VIS spectroscopy, scanning electron microscopy (SEM), energy-dispersive X-ray (EDX) analysis, X-ray powder diffraction (XRD) analysis, and Fourier transform infrared spectroscopy (FT-IR) by following standard procedures in the literature [5].

### UV–VIS spectral analysis

The bio-reduction of Ag^+^ ions to form AgNPs was examined by intermittently measuring the absorption spectra of the reaction solution using a UV–VIS spectrophotometer (Multiskan GO; Thermo Scientific, Waltham, MA, USA) for 24 h at a resolution of 2 nm between 300 nm to 700 nm. The color of the reaction solution was also recorded at each time interval.

### XRD analysis of biosynthesized AgNPs

The nature of biosynthesized AgNPs was determined using XRD (X’Pert MRD; PANalytical, Almelo, The Netherlands), set up at 30 kV and 40 mA with Cu Kα radians at an angle of 2θ. Powdered AgNPs were loaded by even spreading on the sample holder (glass slides) following the procedure described by Iravani et al. [5]; the sample holder has positioned appropriately in the XRD machine and analyzed using the inbuilt software.

### SEM and EDX analyses of biosynthesized AgNPs

The surface morphology and basic composition of the biosynthesized AgNPs were investigated using SEM and EDX analyses. The AgNPs were spread evenly on the sample holder with the help of a carbon tape and sputter-coated with platinum, by means of an ion coater machine for 120 s before observation under SEM (S-4200; Hitachi, Tokyo, Japan). After obtaining SEM images, the elemental composition of AgNPs was investigated using an EDX detector (EDS; EDAX Inc., Mahwah, NJ, USA) connected to the SEM machine, by following the method described in the studies conducted by Patra et al [8] and Zhou et al. [28].

### FT-IR analysis

The FT-IR spectra of the biosynthesized AgNPs and the extract of the outer peel of the AC fruit was obtained using an FT-IR spectrophotometer (Spectrum Two^TM^ FT-IR Spectrometer; PerkinElmer, Waltham, MA, USA) at wavelengths ranging from 400 cm^−1^ to 4000 cm^−1^. A pinch of the pounded sample/2 μL of AC extract was placed at the sample collection point, and analysis was done using an inbuilt software in the computer, which was attached to the instrument. The existence of diverse types of functional groups that contribute to the synthesis of AgNPs was investigated using various modes of vibration by following the methods described by Iravani et al. [5] and Basavegowda et al. [29].

### Antibacterial action of biosynthesized AC-AgNPs

Antibacterial potential of the biosynthesized AC-AgNPs against four different gram-positive foodborne pathogenic bacteria, namely *Bacillus cereus* KCTC 3624, *Listeria monocytogenes* ATCC 19111, *Enterococcus faecium* DB01, and *Staphylococcus aureus* ATCC 13565, was determined using the standard disc diffusion technique [30, 31].

Before analysis, four foodborne pathogenic bacteria were sub-cultured overnight in a nutrient broth medium. Powdered AC-AgNP was dissolved at a concentration of 1000 μg/mL in 5% dimethyl sulfoxide (DMSO) (Sigma-Aldrich, St. Louis, Missouri, USA) and sonicated for 30 min at 30°C to prepare a stock solution. Paper discs (50 μg AC-AgNPs/disc) were prepared by adding 50 μL of the AC-AgNP stock solution to filter paper discs (8 mm; Advantec Toyo Roshi Kaisha Ltd., Tokyo, Japan) and dried for 10 min. To test the antibacterial potential of AC-AgNPs, an overnight culture of pathogenic bacteria (1 × 10^7^ CFU/mL) was evenly spread on previously prepared nutrient agar plates and allowed to dry for 5–10 min. Subsequently, the paper discs were placed on the surface of the plates and incubated for 24 h at 37°C in a bacteriological incubator. The standard antibiotic gentamycin (50 μg/disc) was taken as the positive control and 5% DMSO was taken as the negative control. The diameters of the zones of inhibition around each paper disc were recorded after 24 h of incubation and was considered the antibacterial activity of the sample. Further, the minimum inhibitory concentration (MIC) of AC-AgNPs was determined by the two-fold dilution method with slight variations [30]; [31].

In a 96-well microplate, various dilutions of AC-AgNPs at concentrations of 200, 100, 50, 25, 12.5, and 6.25 μg/mL in 5% DMSO (Sigma-Aldrich, St. Louis, Missouri, USA) were prepared in nutrient broth. Next, 10 μL of the pathogenic cultures were inoculated onto the microplates separately, and the samples were incubated for 24 h at 37°C. The lowermost concentration of AC-AgNPs that did not display any observable growth of the tested bacteria was documented as the MIC value. To determine the minimum bactericidal concentration (MBC), samples were collected from the wells adjacent to the MIC value and the next higher value as well as from the spread cultured in fresh nutrient agar plates and incubated at 37°C for 24 h. The concentration that did not show any growth of a bacterial colony after incubation was considered as MBC.

### Evaluation of cytotoxicity of biosynthesized AC-AgNPs

#### Sample preparation of AC-AgNPs

Dulbecco's phosphate-buffered saline (DPBS; Welgene, Gyeongsanbuk-do, Republic of Korea) was used to dissolve AC-AgNPs at a concentration of 1 mg/mL and disinfected with syringe filters (0.22 μm; Millipore, Billerica, MA, USA). A series of diverse concentrations such as 0.1, 0.01, 0.001, 0.0001, and 0.00001 μg/mL of AC-AgNPs was established using complete Dulbecco’s modified Eagle medium (DMEM; Welgene, Gyeongsanbuk-do, Republic of Korea) supplemented with 10% (v/v) fetal bovine serum (FBS; Gibco, Carlsbad, CA, USA) and 1% (v/v) penicillin–streptomycin (Gibco, Carlsbad, CA, USA) for treating HepG_2_ cells [32].

#### Cell culture and treatment with AC-AgNPs

The HepG2 cell line obtained from the Korean Cell Line Bank (Seoul, Republic of Korea) was cultured in the whole DMEM and maintained at 37°C in a 5% CO_2_ humidified incubator. The fully grown cells were harvested, trypsinized with Trypsin-EDTA (Gibco, Carlsbad, CA, USA), and seeded into 96-well plates at a concentration of 5 × 10^4^ cells per well (100 μL/well). The viability of HepG_2_ cancer cells was 95%, as determined by trypan blue exclusion test. Cells were incubated at 37°C with 5% CO_2_ and 95% air for 24 h in a humidified incubator. After incubation for 24 h, the medium was removed, and the cells were exposed for 24 h to 1, 0.1, 0. 01, and 0. 001, μg/mL of AC-AgNPs that were distributed in complete DMEM. The cells were once again incubated at 37°C with 5% CO_2_ for 24 h which is most suitable for the survival of cancer cells [32].

#### Cell cytotoxicity and viability biosynthesized AC-AgNPs

The cell cytotoxicity of AC-AgNPs was investigated using the EZ-Cytox kit (DoGenBio Co., Ltd., Seoul, Republic of Korea) according to the manufacturer’s guidelines. The optical density of AgNPs suspended in DMEM was scanned in the wavelength from 300–700 nm, before the treatment to HepG2 cell (S1 Fig). The supernatant was replaced after 24 h of exposition, with 110 μL of a fresh complete medium comprising of 10 μL of EZ-Cytox solution and incubated for approximately 20 min (until the melon red color turned to yellowish orange). Subsequently, after incubation, 100 μL of the samples were aliquoted in a new 96-well plate, and the absorbance was noted at 450 nm using a spectrophotometer (Spectra Max 384 Plus; Molecular Devices, Sunnyvale, CA, USA). The viability of cells exposed to AC-AgNP was estimated by performing the trypan blue exclusion assay. Similarly, the supernatant was removed after 24 h of exposition, and the cells were immediately washed with 100 μL of DPBS. Subsequently, 20 μL of fresh complete DMEM and trypan blue mixture (1:1) were added to each well, and cell viability -was observed under an inverted microscope (DMI6000B; Leica, Wetzlar, Germany) [32].

### Antidiabetic potential of biosynthesized AC-AgNPs

The enzyme α-glucosidase (source: *Saccharomyces cerevisiae*; Cat. No. G5003, ≥30 U/mg) and other fine chemicals were purchased from (Sigma-Aldrich, St. Louis, Missouri, USA). The AC-AgNP samples were dissolved in methanol by keeping them in a sonicated water bath for 10–15 mins to attain a final concentration of 10 mg/mL, and the samples were diluted with 0.02 M phosphate buffer (pH 6.9) and used in the assay. Assays for α-glucosidase inhibition were performed by following the protocol standardized in the lab [33]. A total of 10 μg/mL of the samples was aliquoted into 96-well plates and was serially diluted with 0.02 M sodium phosphate buffer (pH 6.9) (Sigma-Aldrich, St. Louis, Missouri, USA), and the volume was maintained at 50 μL. To this, 50 μL of α-glucosidase (0.5 U/mL) was added and incubated for 10 mins at room temperature, followed by addition of 50 μL of 3.0 mM p-nitrophenyl-glucopyranoside (pNPG) as substrate and incubation for 20 mins at 37°C. The reaction was stopped by adding 50 μL of 0.1 M Na_2_CO_3_ (Sigma-Aldrich, St. Louis, Missouri, USA). The absorbance was read at a wavelength of 405 nm using a plate reader. Wells with buffer, substrate, and enzyme were used as positive controls. The percentage inhibition of enzyme activity for α-glucosidase was calculated as follows.

$$\%\;Inhibition=\frac{Abs_{Control}-{\text{Abs}}_{Test}}{Abs_{Control}}\;\times 100$$

### Antioxidant activity of biosynthesized AC-AgNPs

Through three diverse radical scavenging assays, the radical scavenging potential of the biosynthesized AC-AgNPs was confirmed, i.e., reducing power assays, ABTS (2, 2′-azino-bis [3-ethylbenzothiazoline-6-sulphonic acid]) free radical scavenging, and DPPH (1, 1-diphenyl-2-picrylhydrazyl) free radical scavenging assays.

#### ABTS free radical scavenging activity

The ABTS free radical scavenging potential of the biosynthesized AC-AgNPs and butylated hydroxytoluene (BHT) (Sigma-Aldrich, St. Louis, Missouri, USA), taken as the standard reference, was evaluated by following a standard procedure [28]. Initially, stock solutions of 7.4 mM ABTS (Sigma-Aldrich, St. Louis, Missouri, USA) and 2.6 mM potassium persulfate (Sigma-Aldrich, St. Louis, Missouri, USA) were separately prepared. Next, to prepare the ABTS working solution, these stock solutions were mixed in equivalent parts and set aside for 12 h in the dark. During the analysis, in a 96-well microplate, 15 μL of three different concentrations (25–100 μg/mL) of AC-AgNPs or BHT was added to 135 μL of the ABTS working solution and mixed well; these mixtures were then kept for 2 h under dark conditions for the end of the scavenging reaction. Using a UV–VIS spectrophotometer (Multiskan GO; Thermo Scientific, Waltham, MA, USA) with methanol as the reference blank, the absorbance of the reaction mixture was recorded. The scavenging ability of AC-AgNPs and BHT on ABTS free radicals was expressed as follows:

$$\%\;\text{ABTS free radical scavenging effect}=\frac{{\text{C}}_{\text{r}}–{\;\text{T}}_{\text{r}}}{{\text{C}}_{\text{r}}}\times \;100$$

Where C_r_ is the absorbance of the control and T_r_ is the absorbance of the test samples.

#### DPPH free radical scavenging activity

Following the standard procedure described by Zhou et al. [28] and Patra et al. [34], the DPPH free radical scavenging potential of AC-AgNPs and BHT, taken as the reference standard, was evaluated. Reagent mixtures of 0.1 mM DPPH (Sigma-Aldrich, St. Louis, Missouri, USA) in methanol (Honeywell, 64–5 Mugeo-dong, Nam-gu, Ulsan, Korea), and three different concentrations (25–100 μg/mL) of AC-AgNPs or BHT were prepared in methanol prior to the experiment. In a 96-well microplate, the reaction mixture was initiated by adding 50 μL each of DPPH solution and AC-AgNPs or BHT at varied concentrations. The whole mixture was continuously shaken using an orbital shaker under dark conditions for 30 min. The absorbance of the reaction mixture was recorded after the incubation time hour using a UV-VIS spectrophotometer (Multiskan GO; Thermo Scientific, Waltham, MA, USA), with methanol as the reference blank. The scavenging ability of AC-AgNPs and BHT on DPPH free radicals was expressed as follows:

$$\%\;\text{DPPH free radical scavenging effect}=\frac{{\text{C}}_{\text{r}}–{\;\text{T}}_{\text{r}}}{{\text{C}}_{\text{r}}}\times \;100$$

Where C_r_ is the absorbance of the control and T_r_ is the absorbance of the test samples.

#### Reducing power activity

By following a standard procedure described by Zhou et al. [28], the reducing power of the biosynthesized AC-AgNPs was determined. The entire volume of the reaction mixture comprised 50 μL each of AC-AgNPs or BHT in three varying concentrations ranging between 25 and 100 μg/mL, 0.2 M phosphate buffer (pH 6.6), and 1% potassium ferricyanide. The complete mixture was mixed nicely and incubated for 20 min at 50°C in dark condition, and subsequently, 50 μL of 10% trichloroacetic acid (Sigma-Aldrich, St. Louis, Missouri, USA) was added to the entire solution to terminate the reaction. Next, the solution was centrifuged at 3,000 rpm for 10 min, and 50 μL of the supernatant was transferred to a 96-well microplate. Next, 50 μL of double distilled water and 10 μL of 0.1% FeCl_3_ solution were added, and the solution was incubated at room temperature for 10 min. At the end of the experiment, the absorbance of the mixture solution was documented at 700 nm, and the values were taken as the reducing power.

#### Nitric oxide scavenging (NOX) activity

As described previously in the study by Patra et al. [35], AC-AgNPs nitric oxide radical scavenging potential was confirmed. Concisely, a reaction mixture volume of 200 μL was prepared by mixing 100 μL of AgNPs at various concentrations (20 to100 μg/mL) and 100 μL of 10 mM sodium nitroprusside in phosphate buffer saline (pH 7.4). The reaction mixture was then incubated at 37°C for 1 h under light conditions. After incubation, 75 μL aliquots of the reaction mixture were added to 75 μL of Griess reagent (1.0% sulfanilamide and 0.1% naphthyl ethylene diamine dihydrochloride) in a 96-well flat-bottom microplate (SPL Life Sciences, Gyeonggi-do, South Korea), which was incubated under dark conditions for 30 min at 25°C. The absorbance of the reaction mixture was measured using the microplate reader at 546 nm. A reaction mixture composed of 100 μL of methanol and 100 μL of 10 mM sodium nitroprusside in phosphate buffer saline (pH 7.4) was taken as the negative control, whereas a reaction mixture containing 100 μL of different concentrations of BHT (20–100 μg/mL) and 100 μL of 10 mM sodium nitroprusside in phosphate buffer saline (pH 7.4) was taken as the reference control. The nitric oxide scavenging potential of AgNPs was calculated from the following equation:

$$\%\;\text{Nitric oxide scavenging effect}=\frac{{\text{C}}_{\text{r}}–{\;\text{T}}_{\text{r}}}{{\text{C}}_{\text{r}}}\times \;100$$

Where C_r_ is the absorbance of the control and T_r_ is the absorbance of the test samples.

### Statistical analysis

All data are expressed as the mean value of three independent replicates ± the standard deviation (SD). Statistical analysis was carried out by one-way analysis of variance (ANOVA) test followed by Duncan’s multiple range test using SPSS software, version 23.0 (IBM Corp., Armonk, NY, USA) at a 5% level of significance (*P* < 0.05).

## Results

### Synthesis of AgNPs using AC extract

**Fig 1:**
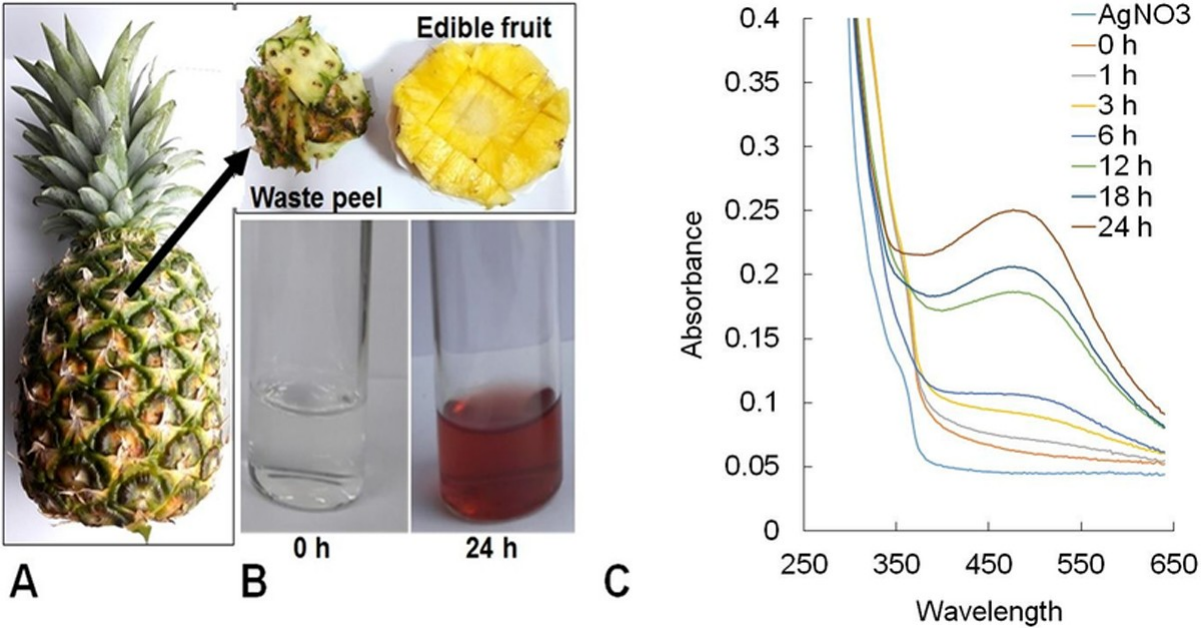
(A) *Ananas comosus* (AC) fruit and waste product (B). A gradual change in the color of AC extract during the synthesis of AC-AgNPs between 0 and 24 h. (C) UV–VIS spectral analysis of the biosynthesized AC-AgNPs.

In this current study, biosynthesis of AC-AgNPs was carried out under laboratory condition using the outer peel of AC fruit (Fig 1A), which is basically a food waste material (Fig 1A). The synthesis seemed progressive as confirmed by the change in the color of the reaction medium from colorless to reddish-brown (Fig 1B).

### Characterization of biosynthesized AC-AgNPs

**Fig 2:**
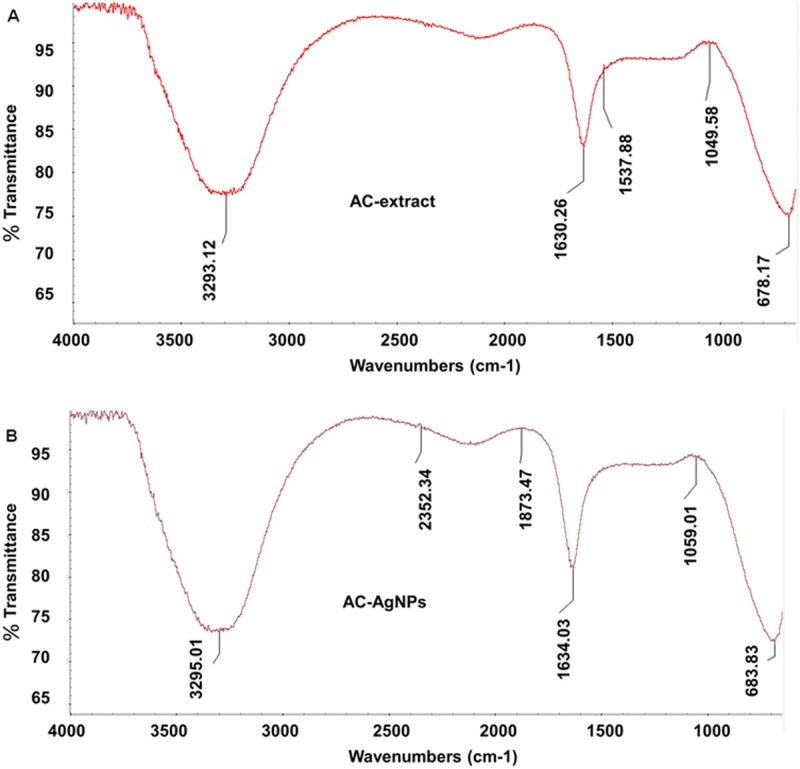
FT-IR analysis of (A) AC extract and (B) AC-AgNPs.

After visual confirmation by detecting a color change in the biosynthesis of AC-AgNPs, the samples were exposed to spectral analysis. The UV–VIS spectra of the reaction mixture were recorded for 24 h at varied time intervals. The highest absorbance peak of the solution mixture was detected at 485 nm in case of AC-AgNPs (Fig 1C). Further, the AC extract and AC-AgNPs were subjected to FT-IR analysis, and the results are presented in Fig 2. Absorption peaks were detected at 3293.12, 1630.26, 1537.88, 1049.58, and 678.17 cm^**−1**^ for the AC extract (Fig 2A), and at 3295.01, 2352.34, 1873.47, 1634.03, 1059.01, and 683.83 cm^**−1**^ for the AC-AgNPs (Fig 2B). As per the result of the UV–VIS spectra and FT-IR analysis, AC-AgNPs were established to be very stable and hence used in subsequent studies.

**Fig 3:**
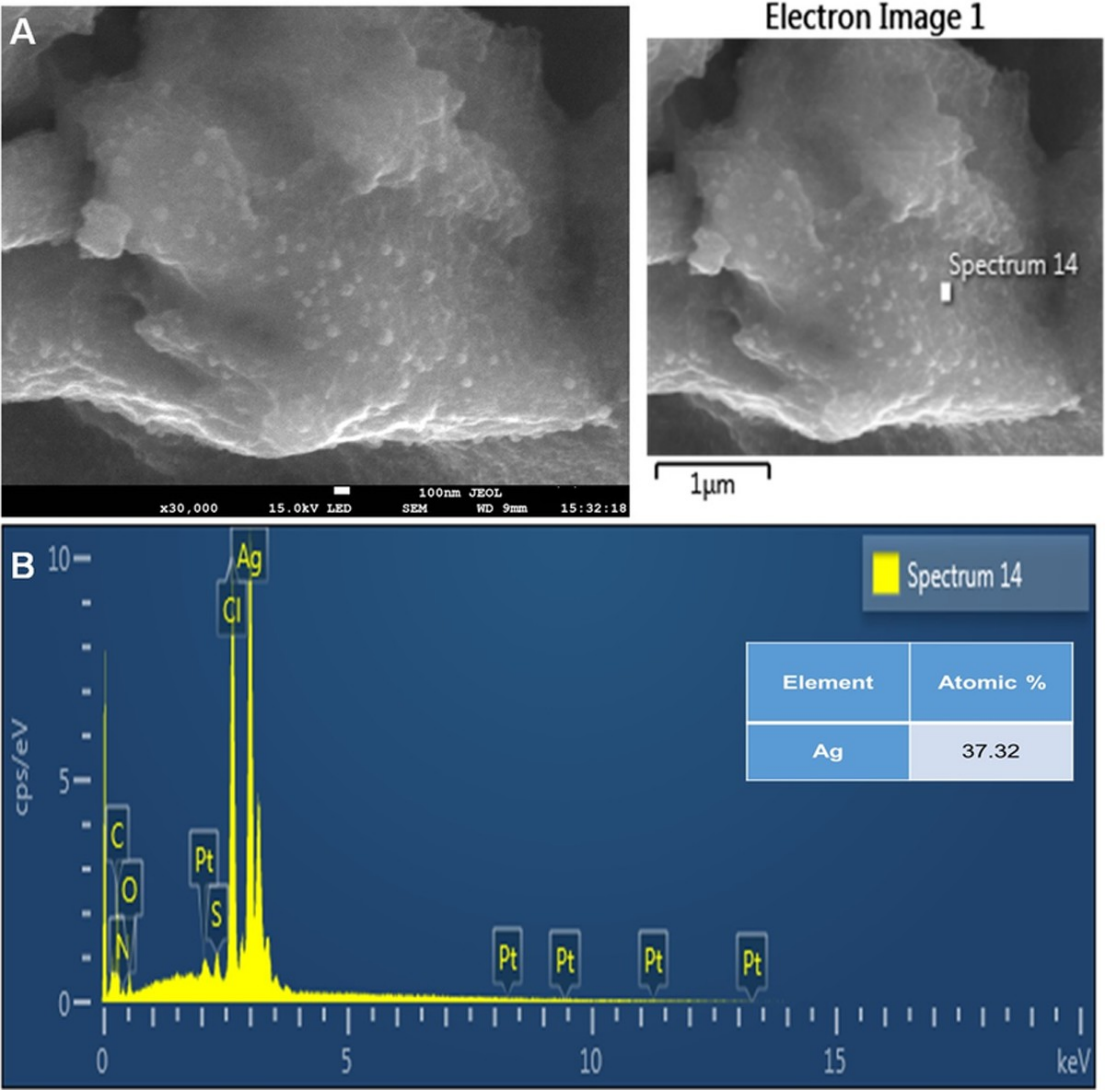
(A) SEM and (B) EDX spectral analysis of the biosynthesized AC-AgNPs.

**Fig 4:**
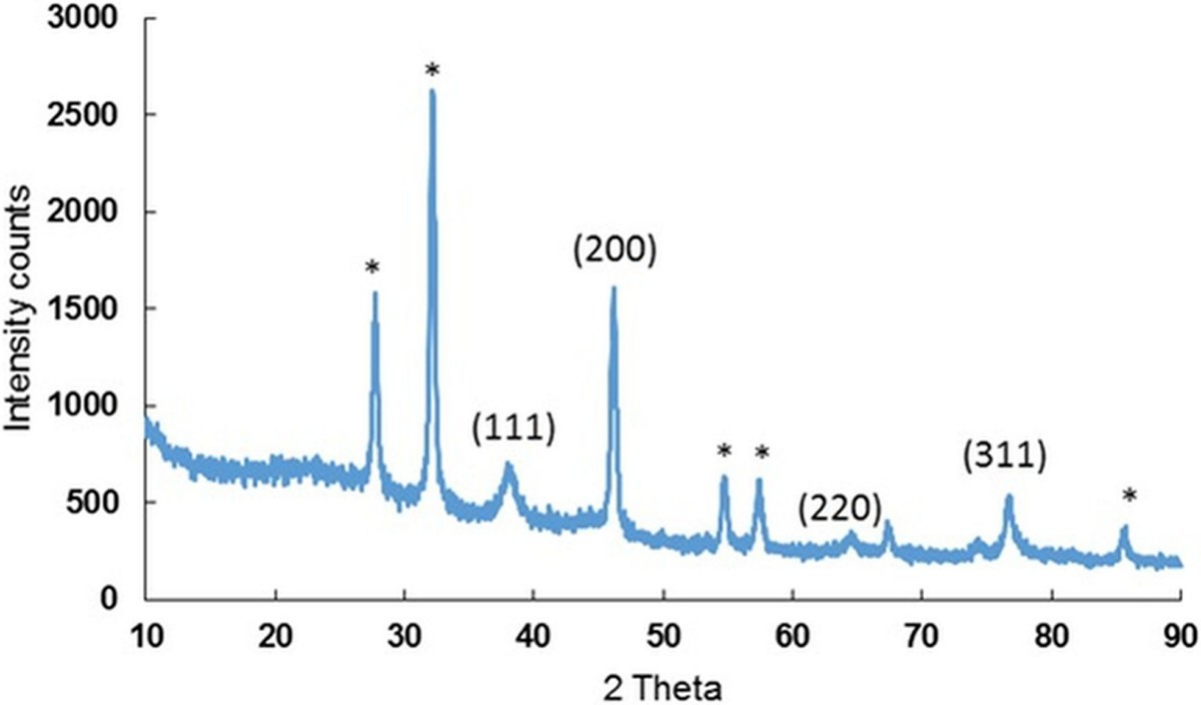
XRD analysis of the biosynthesized AC-AgNPs.

The basic structure and morphology of the biosynthesized AC-AgNPs were studied via SEM-EDX analysis. The results of surface morphology revealed that the AC-nanoparticles were clustered in nature with nearly spherical structures (Fig 3A). By means of the EDX analysis, with a detector connected to the SEM machine, the elemental configuration of the biosynthesized AC-AgNPs was established (Fig 3B). The plotted graph exhibited a strong peak at 3 keV, conforming to the Ag element, thereby specifying the existence of AgNPs. The Ag element accounted for 37.32% of the total composition (Fig 3B). EDX analysis also revealed the presence of other elements such as Cl, O, N, Pt, and S (Fig 3B). The XRD configuration of the biosynthesized AC-AgNPs is shown in Fig 4. The diffraction configuration showed four well-resolved diffraction peaks at 2θ angles of 38.14, 46.13, 64.67 and 76.72 corresponding to (111), (200), (220) and (311) respectively (Fig 4).

### Bio-potential of the biosynthesized AC-AgNPs

The characteristics of the biosynthesized AC-AgNPs were evaluated in terms of their antioxidative, antibacterial, antidiabetic, and cytotoxic potential. The antibacterial property of AC-AgNPs was determined against four different foodborne pathogenic bacteria, namely *Enterococcus faecium* DB01, *Listeria monocytogenes* ATCC 19111, *Bacillus cereus* KCTC 3624, and *Staphylococcus aureus* ATCC 13565 (Table 1). The results of the antibacterial activity revealed that AC-AgNPs were active against the four above mentioned foodborne pathogenic bacteria and exhibited inhibition zones with diameters ranging between 8.78–10.31 mm (Table 1 and S2 Fig). Gentamycin, which was used as a positive control, demonstrated inhibition zones, measuring 9.01–15.82 mm in size, against each of the tested bacteria. In addition, the MIC value of AC-AgNPs against all the four foodborne pathogenic bacteria was in the range of 50–100 μg/mL, and the MBC values were 100 - >100 μg/mL.

**Table 1:** Antibacterial activity of AC-AgNPs against foodborne pathogenic bacteria.

Pathogenic bacteria	AC-AgNPs*	Positive control**	Negative control***	MIC^#^	MBC^#^
*E*. *faecium*	10.31^c^ ±0.68	9.01^d^ ±0.56	0±0	50	100
*L*. *monocytogenes*	9.07^d^ ±0.11	10.99^bc^ ±0.48	0±0	50	100
*B*. *cereus*	8.91^d^ ±0.05	15.82^a^ ±0.30	0±0	100	>100
*S*. *aureus*	8.78^d^ ±0.08	11.09^b^±0.47	0±0	100	>100

*AC-AgNPs at 50 μg/disc;

**Positive control—Gentamycin (50 μg/disc);

***Negative control– 5% DMSO;

“#”–values in μg/mL. All data are presented in terms of the diameter of zones of inhibition in mm. The difference in the superscript letters in each column is significant at *P* < 0.05.

**Fig 5:**
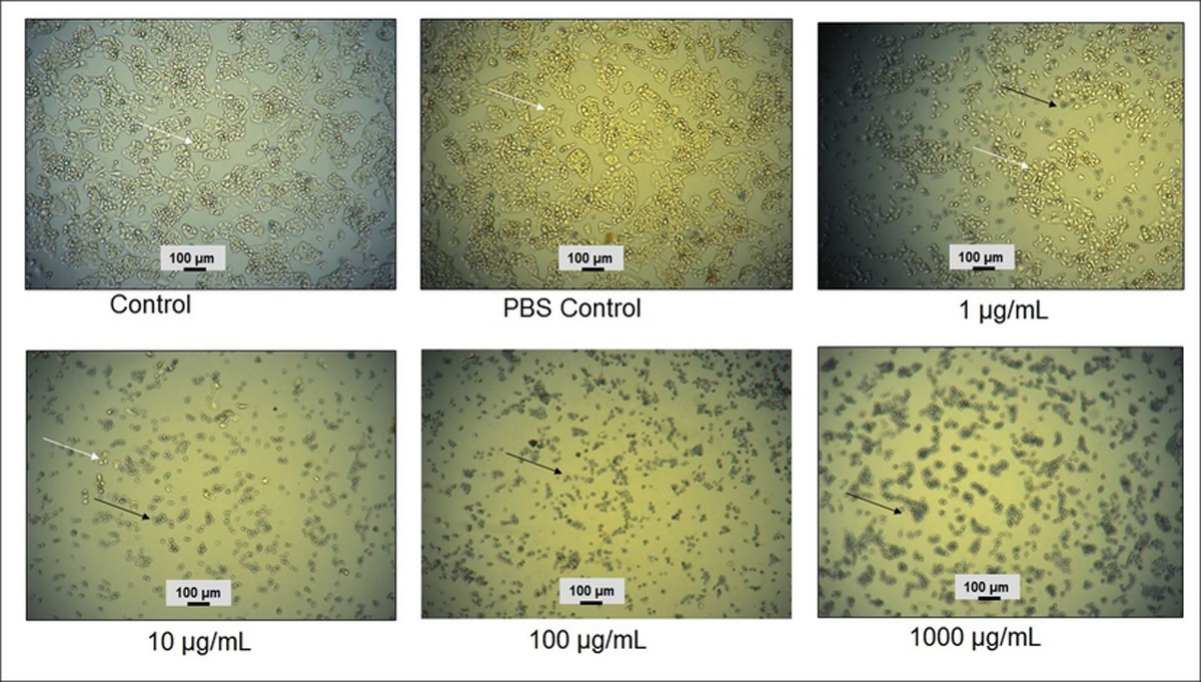
Cytotoxicity activity (effect of treatment of AC-AgNPs with HepG_2_ cancer cells: Black arrow indicating dead cells and a white arrow indicating live cells.

Substantial variations signifying the cell death as well as limited scattering configurations and the larger number of dead cells (black arrow) were detected at higher AC-AgNPs concentrations (Fig 5), whereas more number of even spreading pattern alive cells (white arrow) were detected at lower concentrations (Fig 5). The image of control cells demonstrated that there was a greater number of alive and well-attached cells (Fig 5). When the treated HepG_2_ cancerous cells were observed under an inverted microscope, it was observed that AC-AgNPs were highly toxic to HepG_2_ cancer cells at a higher concentration. The cytotoxicity of AC-AgNPs against HepG_2_ cancer cells was evaluated after 24 h of exposure.

**Fig 6:**
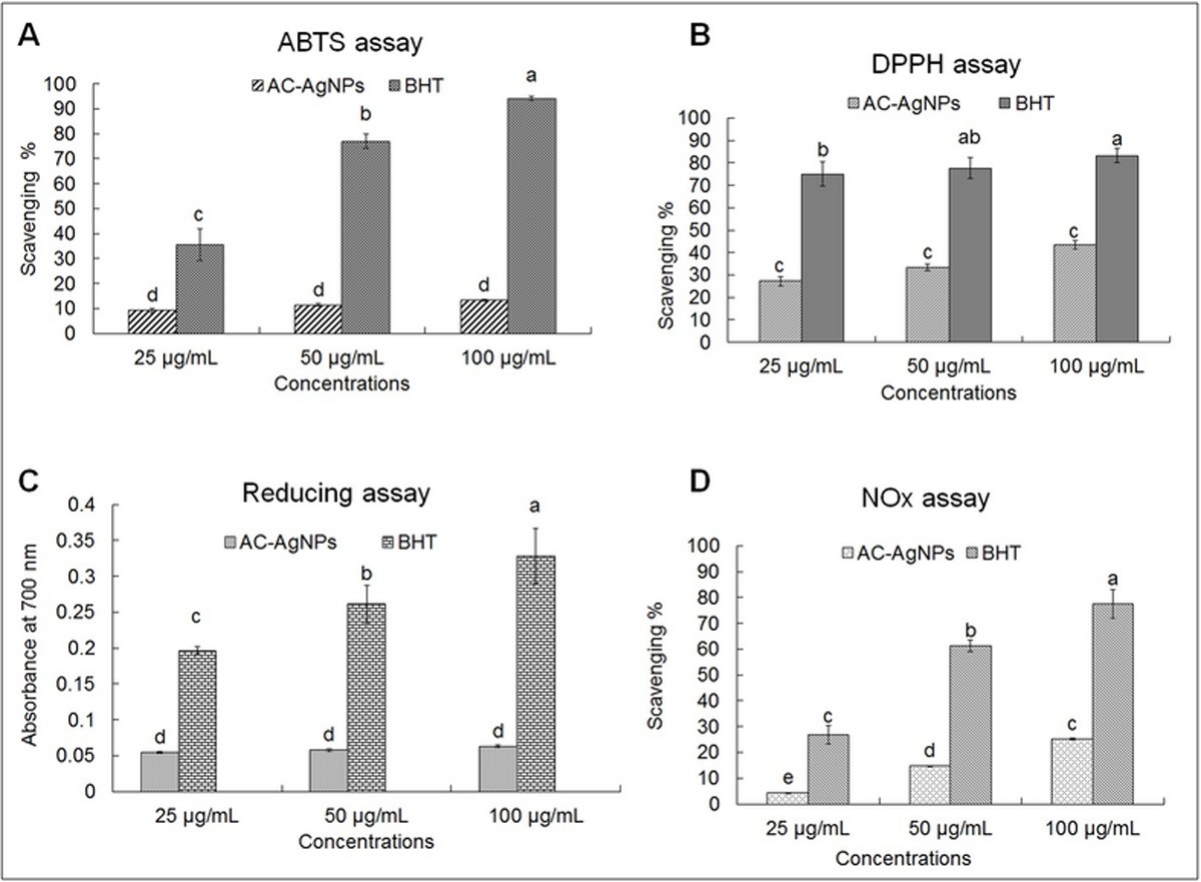
Antioxidant scavenging potential of the biosynthesized AC-AgNPs. (A) ABTS radical scavenging activity; (B) DPPH free radical scavenging activity; (C) Reducing power assay and; (D) NOX assay of AC-AgNPs. Different superscript letters in each column of each figure indicate statistical significance (*P* < 0.05).

The biosynthesized AC-AgNPs were further investigated for their antioxidative potential according to the assays performed in various radical scavenging studies, i.e., the ABTS free radical scavenging, DPPH free radical scavenging, and reducing power assays; the results are displayed in Fig 6. The ABTS radical scavenging activity of AC-AgNPs at the three different concentrations was in the range of 9.18%–13.32%, whereas that of BHT was in the range of 35.57%–94.07% (Fig 6A). The DPPH free radical scavenging activity of AC-AgNPs at a concentration of 25–100 μg/mL ranged from 27.23% to 43.41% (Fig 6B), whereas that of BHT ranged from 75.04% to 83.31% at the same concentration. The reducing powers of AC-AgNPs and BHT at concentrations of 25–100 μg/mL were in the range from 0.054–0.063 and 0.196–0.328, respectively (Fig 6C). However, the NOX result of the AC-AgNPs and BHT at concentrations of 25–100 μg/mL was in the range of 4.28–25.25 and 26.91–77.4, respectively (Fig 6D).

**Fig 7:**
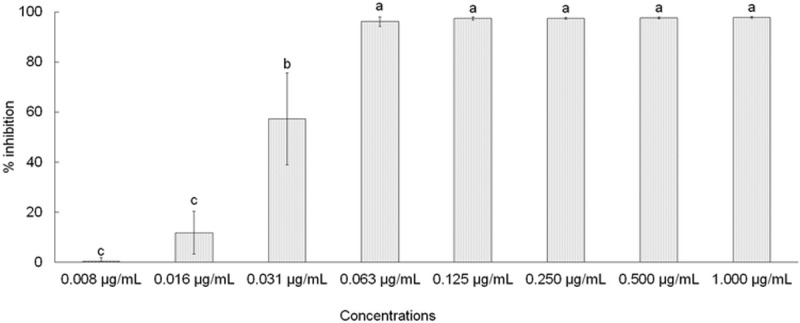
Alpha-glucosidase assay of AC-AgNPs. Different superscript letters in each column indicate statistical significance (*P* < 0.05).

The assessment of α-glucosidase effects of AC-AgNPs revealed a promising antidiabetic potential of the AC silver NPs in terms of its α-glucosidase inhibition effect (Fig 7). AC-AgNPs at concentrations of 0.063, 0.125, 0.250, 0.500, and 1.000 μg/mL showed a 100% inhibition and were highly effective.

## Discussion

The use of physical and chemical synthesis methods are often avoided in the synthesis of AgNPs due to the use of toxic chemicals and toxic byproducts which are both toxic to the environment and also high cost and are not useable for biomedical applications. Recently, the green synthesis of metal and metal oxide NPs have attracted generous attention in nano-research, because of their unique optical, electrical, catalytic and magnetic and non-toxic nature along with the environmentally friendly approach, cost-effectiveness, and suitability for use in the biomedical field [36, 37]. Silver nanoparticles are useful in food industries, agriculture, biomedical settings, drug delivery, etc. [38, 39]. To avoid any harmful environmental impact, the green nanoparticle synthesis-based approaches were devoid of any toxic chemicals in sample preparation and synthesis procedures.

Rules governing organic solid waste management and ecological worries have been increasing. Hence, from fruit wastes, such as fruit peels, and other organic fractions of domestic solid wastes may be used in an effective manner in nanotechnology-based applications [6, 39, 40]. The peels of the different fruits contain several natural active compounds like flavonoids, phenolic compounds, essential oils, and natural color [17, 18]. In the current study, for the green synthesis of AC-AgNPs, the outer peel of the AC fruit extract is used (Fig 1A). The biosynthesis of AC-AgNPs was initially determined by observing the change in the reaction mixture from a transparent to a reddish-brown color. The gradual color change in the reaction mixture indicated the formation of AC-AgNPs (Fig 1B) [41, 42]. The previously published articles report that AC contains various beneficial phytochemicals, which have anti-cancer, antioxidative and antibacterial potential [9, 17, 18]. These compounds having biological properties such as antimicrobial, antioxidative, anti-proliferative, and anti-inflammatory activities, which can be used in pharmacological, hygiene, biomedical, or pharmaceutical applications [43]. Consequently, the outer peel extract of AC might have the same above mentioned bioactive compounds, and these compounds could be responsible for the bioreduction and various active potential effects of the synthesized AC-AgNPs.

After the green-synthesis of AC-AgNPs, the reaction kinetics was initially monitored by UV–VIS absorption spectroscopy in the wavelength range of 350–650 nm. Usually, due to the excitation of free electrons, AgNPs would display a surface plasmon resonance (SPR) band at 450–550 nm [42, 44]. In the current study, the SPR value of AC-AgNPs was detected at 485 nm (Fig 1C). The current result is similar to the previously reported AgNPs synthesis result [45].

The FT-IR analysis, identifies the functional groups exist in the sample which are the responsible capping agents in the stabilization of the biosynthesized AgNPs [46]. The FTIR result of this study displayed five different stretching bonds in the AC extract (Fig 2A) and six different stretching bonds in the AC-AgNPs (Fig 2B). It is expected that the peaks at (3293.12, 1630.26, 1049.58, and 678.17 cm^**−1**^) in AC extract (Fig 2A) shifted to higher wavenumbers 3295.01, 1634.03, 1059.01, and 683.83 cm^**−1**^ respectively in case of AC-AgNPs (Fig 2B). The alterations in the stretching bonds of the AC-AgNPs could be attributed to the reduction, capping, and stabilization process during AgNPs synthesis [41, 42]. In AC-AgNPs the peaks at 3295.01 cm^**−**1^ specifies the existence of strong broad vibrations of O–H stretch, H–bonded bond which belongs to the alcohols and phenols functional group [47, 48]. Similarly, the peak at 1634.03 cm^**−**1^ indicates the existence of N-H bend, which belongs to amine functional group, the peak 1059.01 cm^**−**1^ (indicates the existence of C–O stretch) it belongs to the alcohols, carboxylic acids, esters, ethers functional group. Another peak observed at 683.83 cm^**−**1^ (indicates the existence of the C–H bond belongs to aromatics functional group [47, 48]. In various studies, it is stated that these functional groups have an active role in the capping/stabilization of AgNPs [49–52]. The aqueous AC extract comprised of flavonoids, phenolic acids and various functional groups counting carboxyl, ketones, aldehydes, and carboxyl groups, which act as capping and stabilizing agent, and reduces silver ion to Ag^0^ in the synthesis process of AC-AgNPs from AC-extract as evident from previous studies [51–55]. Analogous results were reported in prior studies [56, 57].

SEM analysis displayed the nearly spherical shape of the AC-AgNPs in the nanometer range; however, these were agglomerated in some cases (Fig 3A) during the synthesis process, which could be attributed to solvent vanishing [42]. However, the nanoparticles were dispersed uniformly when mixed with respective solvents for various assays. The EDX result reveals the elemental configuration and the atomic percentage of Ag is 37.32% with some other elements in the biosynthesized AC-AgNPs, which can be credited to the plant variety used in the synthesis procedure, whereas the flavonoids and proteins in the plant material might be involved in the capping of AC-AgNPs [42]. The greater percentage of Ag, as confirmed by elemental investigation, established that the particles were mostly composed of Ag (Fig 3B). EDX analysis also revealed the presence of other elements (Fig 3B), which could have originated from the biomolecules and assisted as organic capping agents which are bound to the exterior of the AgNPs. [58, 59]. Besides, the element Pt was also detected in the EDX spectra, which was due to the reason of using Pt ion for coating the synthesized AgNPs while preparing the sample for the AgNPs analysis in the SEM machine.

XRD analysis revealed the presence of four distinct peaks (Fig 4). These (peaks) are the characteristic of the metallic face-centered cubic (fcc) phase of Ag and matching with the database base of -standard (JCPDS Card no. 04–0783) [42, 60] and confirmed the crystalline nature of the biosynthesized AC-AgNPs. Same values were stated earlier [41, 42]. Besides, few unassigned peaks were also observed that suggests that crystallization of the bio-organic phase might have occurred on the surface of the synthesized AC-AgNPs or might be due to phytoconstituents in the extract involved in the biosynthesis and stabilization process of silver NPs [46, 61].

After the characterization of biosynthesized AC-AgNPs, the antibacterial action, cytotoxic potential, antidiabetic potential, and free radical scavenging activity, of the AC-AgNPs were evaluated. At present, the growth of multidrug-resistant pathogens is prevalent, which can potentially have a negative effect on human health [62]. Because of this concern, the antibacterial action of AC-AgNPs against pathogenic bacteria has been studied. The positive results of AC-AgNPs against gram-positive pathogenic bacteria in this study are listed in Table 1; these positive results can be attributed to the smaller size of the AC-AgNPs, which might enable the entry of these nanoparticles into pathogenic bacterial cells and consequent destruction of bacterial proteins; this will, in turn, result in the bursting of cells and kill the bacteria [63]. It is also possible that the antibacterial activity displayed in the current study might be due to the dual effect of AgNPs and Ag_2_O/AgO [9, 64]. In the previous research, there is also reports of antimicrobial activity of AC peel and other fruit peel extract AgNPs [9, 65]. The AC-AgNPs displayed a MIC/MBC value in the range of 50–100 μg/mL, which further strengthen the potential effect of the NPs against the pathogenic bacteria and their applications in various fields such as wound dressing, antibacterial coatings etc.

The possible cytotoxic activity of AC-AgNPs against HepG_2_ cancer cells was also validated (Fig 5); it was found to be higher with increasing concentration of AC-AgNPs. The cytotoxic activity of AC-AgNPs may be attributed to a number of possible reasons such as the induction of cellular damage, initiation of different immunological reactions and electrostatic attraction between the treated cells and the AC-AgNPs [32, 66–68]. The result indicates that AC-AgNPs at lower concentration were also capable to hinder cancer cell growth. Additionally, similar results were also shown by previously published articles, where biosynthesized AgNPs exhibited cytotoxicity potential [8, 44, 67, 69–71].

The biosynthesized AC-AgNPs were evaluated for their free radical scavenging potential. The results revealed a moderate ABTS, reducing, and NOX scavenging activity (Fig 6A, 6C and 6D) of AC-AgNPs, which can be attributed to the interference of several functional groups in the AC extract that are involved in the capping and stabilization procedure on the surface of AC-AgNPs [72]. Within 30 min of reaction, the AC-AgNPs exhibited higher scavenging activity for DPPH assay (Fig 6B) than the ABTS, reducing and NOX assay at 25–100 μg/mL concentrations; this can be credited to the integration of additional oxidants onto the surface of AC-AgNPs owing to a large surface zone [42]. The reducing action of AC-AgNPs (Fig 6C) might be credited to the existence of phenolic functional groups on the surface. In the nervous system, cardiovascular system, and the immune system, nitric oxide is a vital bio-regulatory molecule [73]. The enduring manifestation of nitric oxide is related to a number of diseases including cancers, inflammation conditions and arthritis [74]. Additional, the highly reactive nitric oxide becomes more sensitive when it comes in contact with oxygen and thus produces highly reactive molecules/compounds that can cause a number of cellular aliments like fragmentation of cellular DNA fragmentation and peroxidation of lipids [75]. The NOx activity of the AC-AgNPs could be attributed to the functional compounds in the AC extract that has acted in the capping and stabilization of AC-AgNPs and which could have compete with the oxygen to react with the nitric oxide and could have inhibited the generation of nitrite [76, 77].

AC-AgNPs exhibited promising antidiabetic potential in a dose-dependent manner (Fig 7). Almost 100% inhibition of α-glucosidase was observed at a concentration of as low as 1 μg/mL. A similar effect of AgNPs against α-glucosidase has also been reported in previous publications [78]. It has been known that the inhibition of α-glucosidase digestive enzymes is precisely useful for the treatment of non-insulin diabetes because it will slow down the release of glucose in the blood [78, 79]. The high activity of AC-AgNPs against α-glucosidase observed in the current investigation is a positive indication of its utility in diabetes treatment.

## Conclusions

AC-AgNPs were found to be effectively biosynthesized using the outer peel extract of the AC fruit by employing a green nanotechnology method. The synthesis process was eco-friendly and cost-effective as it involves the use of food waste materials. The current procedure of using food waste materials could be an effective tool in waste management and the development of renewable resources. The higher content of flavonoids and phenolic acids existing in the AC peel extract could have supported in the fast reduction of Ag+ ions and better stabilization of AC-AgNPs. The biosynthesized AC-AgNPs were crystalline in nature as evident from the XRD spectral analysis. The SEM and EDX analysis also confirmed the formation of AgNPs. Further, the AC-AgNPs exhibited high anti-diabetic potential with higher cytotoxic activity against the HepG_2_ cancer cell lines in a dose-dependent manner. It also exhibited moderate antioxidant and antibacterial activity. The results of the current study suggested that AC-AgNPs can prove to be an effective component in various biomedical applications including the management of serious diseases such as diabetes, cancer followed by its uses in antibacterial wound dressings as an antibacterial agent.

## Supporting information

S1 Fig Optical density of AgNPs suspended in DMEM before the treatment to HepG_2_ cell. (DOCX)

S2 Fig Antibacterial activity of (A) Standard positive control, gentamycin and (B) AC-AgNPs against the pathogenic bacteria.
(DOCX)

S1 File Supporting raw data. (DOCX)
