# Supplementary figures and images for: Investigation of antioxidant, antibacterial, antidiabetic, and cytotoxicity potential of silver nanoparticles synthesized using the outer peel extract of Ananas comosus (L.)
Source: PLoS One. 2019 Aug 12;14(8):e0220950. doi: 10.1371/journal.pone.0220950 (PMC6690543; doi:10.1371/journal.pone.0220950)

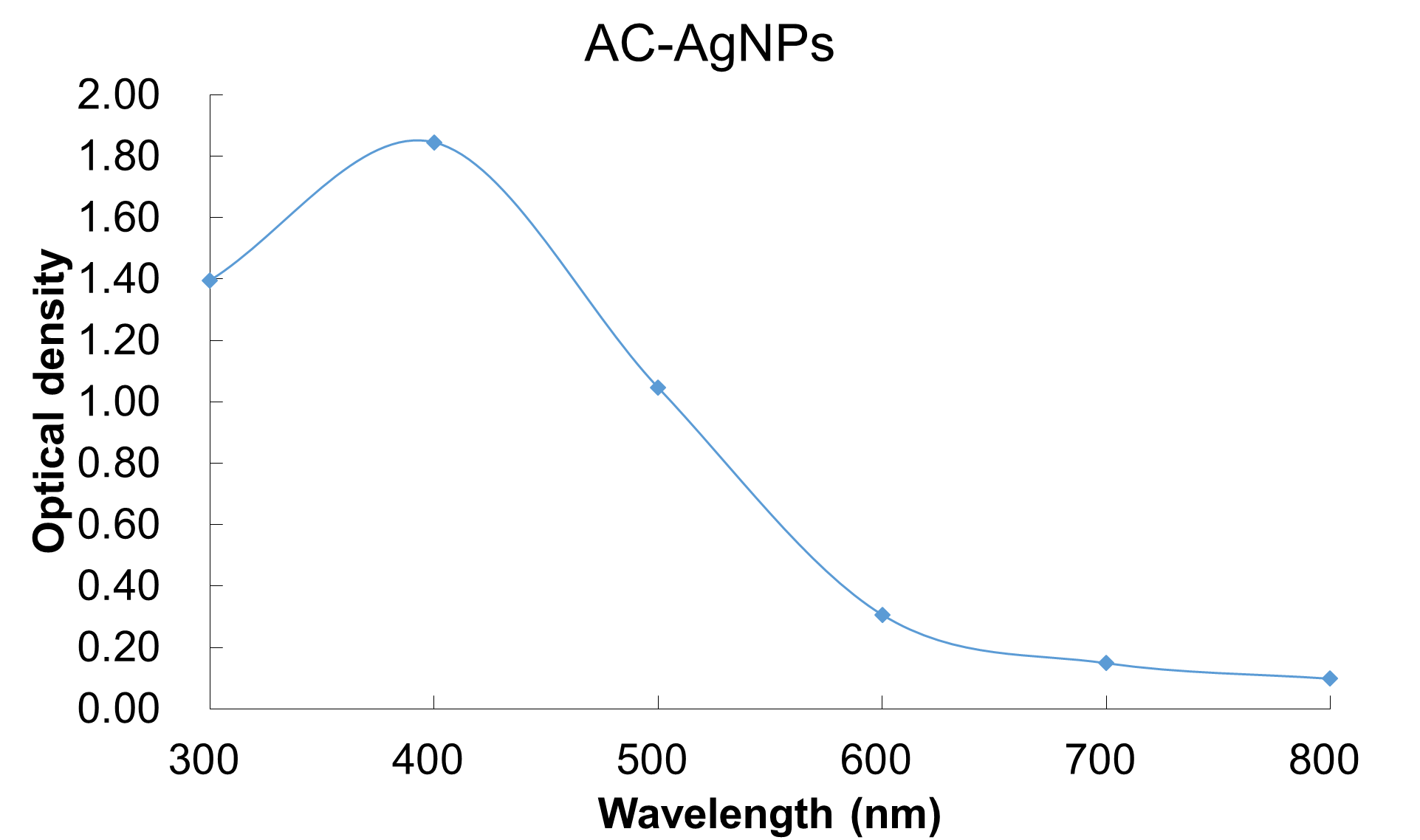


**S1 Fig: Optical density of AgNPs suspended in DMEM before the treatment to HepG2 cell.**

Supplement: S1 Fig — (DOCX) [file pone.0220950.s001.docx]
