# Supplementary material for: Investigation of antioxidant, antibacterial, antidiabetic, and cytotoxicity potential of silver nanoparticles synthesized using the outer peel extract of Ananas comosus (L.)
Source: PLoS One. 2019 Aug 12;14(8):e0220950. doi: 10.1371/journal.pone.0220950 (PMC6690543; doi:10.1371/journal.pone.0220950)
Supplement: S2 Fig — Antibacterial activity of (A) Standard positive control, gentamycin and (B) AC-AgNPs against the pathogenic bacteria. (DOCX) [file pone.0220950.s002.docx]

**
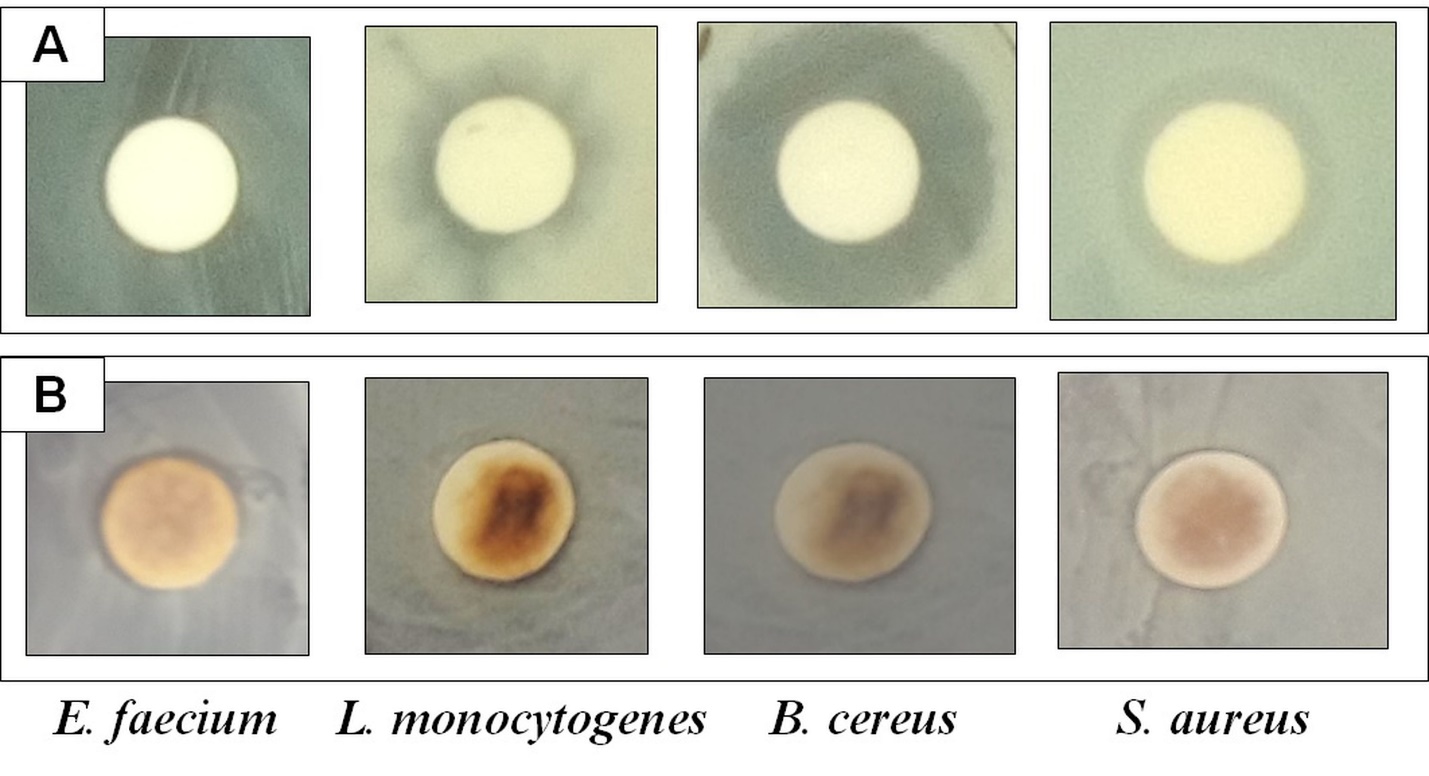
S2 Fig: Antibacterial activity of (A) Standard positive control, gentamycin and (B) AC-AgNPs against the pathogenic bacteria.**
