# Supplementary material for: Investigation of antioxidant, antibacterial, antidiabetic, and cytotoxicity potential of silver nanoparticles synthesized using the outer peel extract of Ananas comosus (L.)
Source: PLoS One. 2019 Aug 12;14(8):e0220950. doi: 10.1371/journal.pone.0220950 (PMC6690543; doi:10.1371/journal.pone.0220950)
Supplement: S1 File — (DOCX) [file pone.0220950.s003.docx]

**Supporting raw data**

**Investigation of antioxidant, antibacterial, antidiabetic, and cytotoxicity potential of silver nanoparticles synthesized using the outer peel extract of *Ananas comosus*(L.)**

1. SEM and EDX data


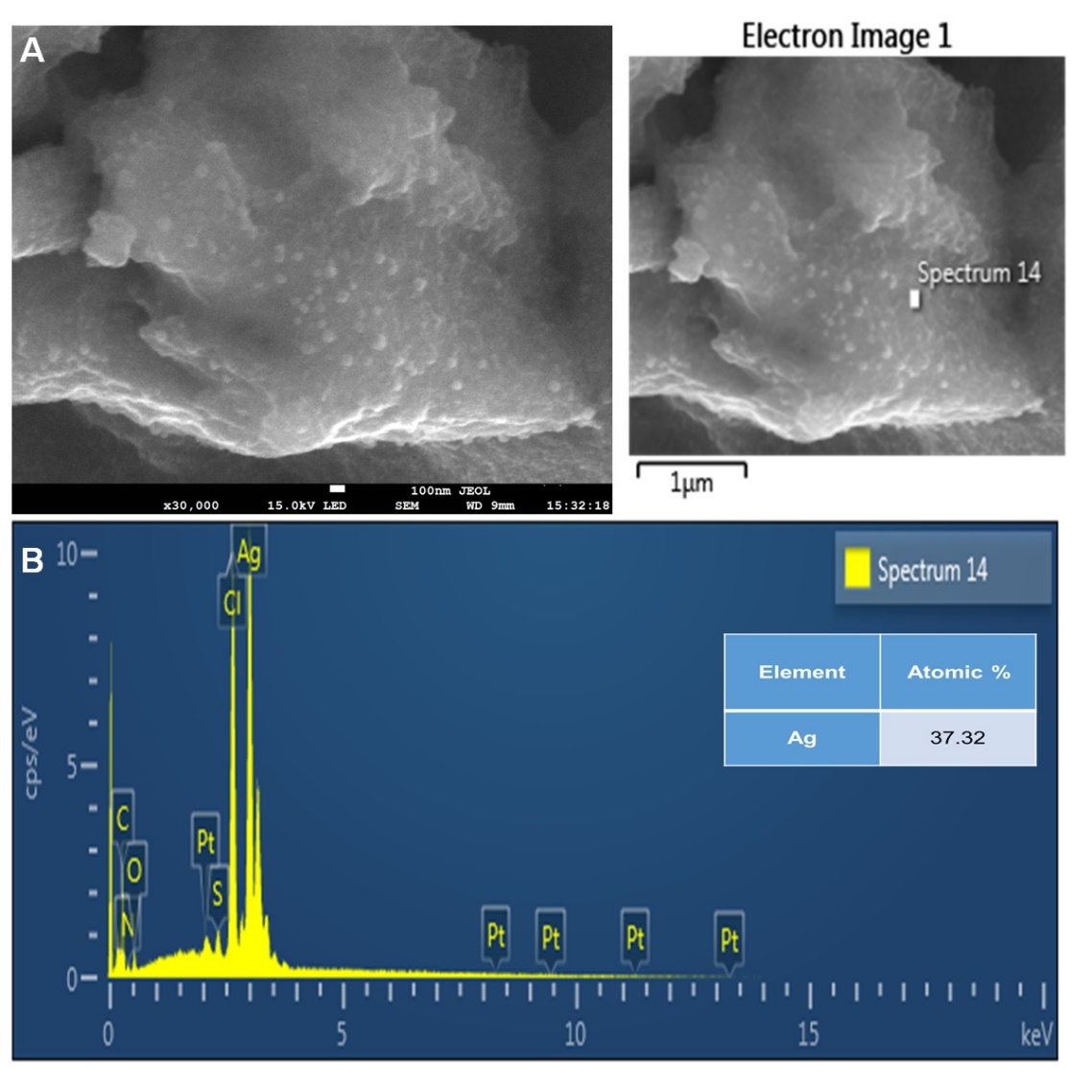


1. XRD data provided in Excellsheet.
2. FTIR data


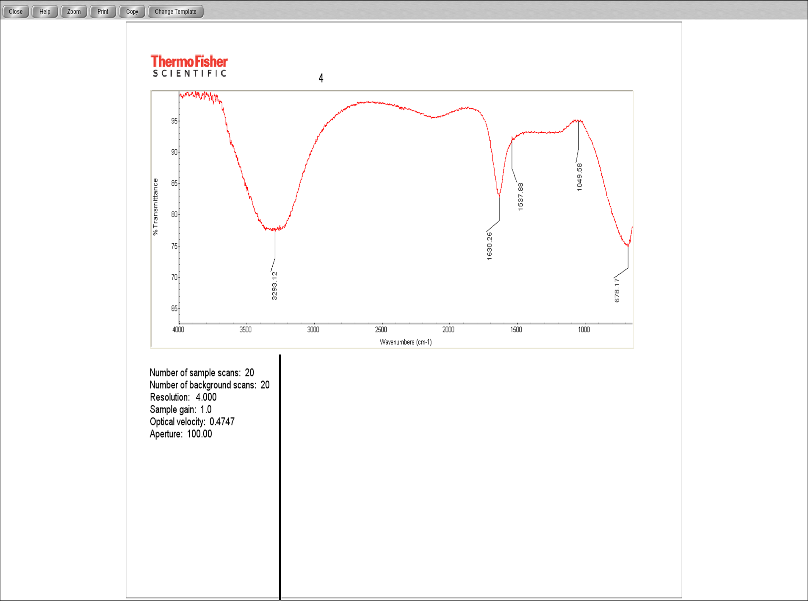


AC-extract


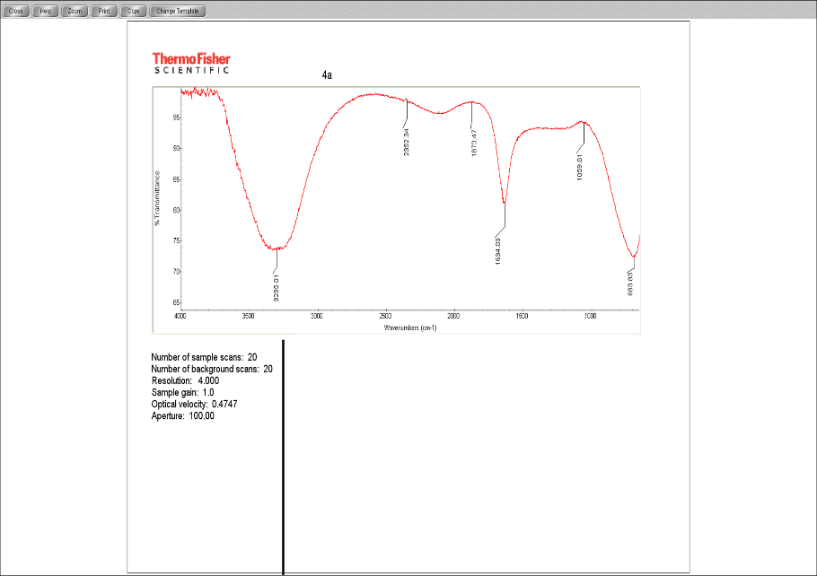


AC-AgNPs

1. **Antibacterial data (raw data)**

| SL.NO. | AC AgNPs | | |  |  |
| --- | --- | --- | --- | --- | --- |
|  | inhibition zone in mm | | | mean | sd |
| *Enterococcus faecium* DB01 | 9.49 | 10.46 | 10.98 | 10.31 | 0.68 |
| *Listeria monocytogenes* ATCC 19111 | 9.14 | 9.15 | 8.93 | 9.07 | 0.11 |
| *Bacillus cereus* KCTC 3624 | 8.91 | 8.86 | 8.97 | 8.91 | 0.05 |
| *Staphylococcus aureus* ATCC 13565 | 8.69 | 8.84 | 8.83 | 8.78 | 0.08 |
|  |  |  |  |  |  |

**Antibacterial figure**


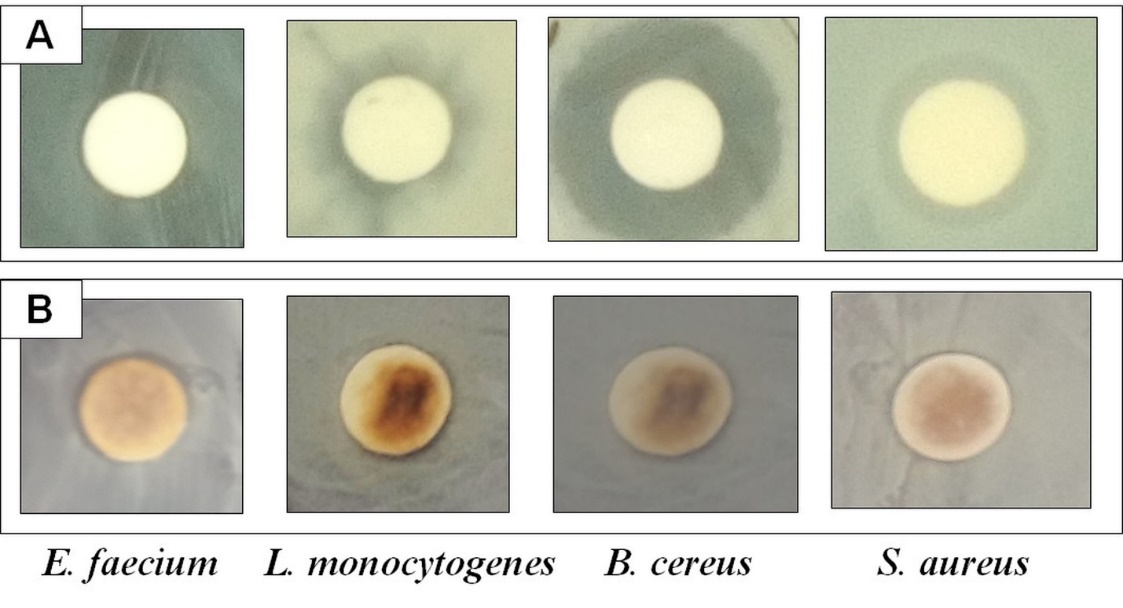


**Supplementary Fig 2: Antibacterial activity of (A) Standard positive control (gentamycin) and (B) AC-AgNPs against the pathogenic bacteria.**

1. **Antioxidant data (raw data)**

|  |  |  | **ABTS** |  |  |
| --- | --- | --- | --- | --- | --- |
|  | Percentage |  |  |  |  |
|  | conc | T1 | T2 | T3 | mean |
| AC-AgNPs | 25 µg/ml | 8.31 | 9.49 | 9.75 | 9.18 |
|  | 50 µg/ml | 10.96 | 11.27 | 12.03 | 11.42 |
|  | 100 µg/ml | 13.05 | 13.08 | 13.84 | 13.32 |
|  | Percentage | | | | |
|  | Conc | T1 | T2 | T3 | mean |
| BHT | 25 µg/ml | 28.48 | 41.11 | 37.14 | 35.57 |
|  | 50 µg/ml | 73.98 | 77.36 | 79.71 | 77.01 |
|  | 100 µg/ml | 93.71 | 94.98 | 93.52 | 94.07 |

|  |  |  | **DPPH** |  |  |
| --- | --- | --- | --- | --- | --- |
|  |  |  | Percentage |  |  |
|  | conc | T1 | T2 | T3 | mean |
| AC-AgNPs | 25 µg/mL | 25.44 | 29.44 | 26.81 | 27.23 |
|  | 50 µg/mL | 31.86 | 33.64 | 34.70 | 33.40 |
|  | 100 µg/mL | 41.44 | 43.54 | 45.22 | 43.40 |
|  | Percentage | | | | |
| BHT | 25 µg/mL | 72.34 | 71.5 | 81.28 | 75.04 |
|  | 50 µg/mL | 82.64 | 73.39 | 77.18 | 77.74 |
|  | 100 µg/mL | 79.81 | 84.43 | 85.69 | 83.31 |

|  |  |  | **Reducing** |  |  |
| --- | --- | --- | --- | --- | --- |
|  |  | T1 | T2 | T3 | mean |
| AC-AgNPs | 25 µg/mL | 0.0557 | 0.0539 | 0.0548 | 0.0548 |
|  | 50 µg/mL | 0.0577 | 0.0595 | 0.0558 | 0.0576 |
|  | 100 µg/mL | 0.0634 | 0.0645 | 0.0617 | 0.0632 |
|  |  |  |  |  |  |
| BHT | 25 µg/mL | 0.1968 | 0.2018 | 0.1911 | 0.1965 |
|  | 50 µg/mL | 0.2896 | 0.2569 | 0.2376 | 0.2613 |
|  | 100 µg/mL | 0.3498 | 0.2839 | 0.3515 | 0.3284 |

|  |  |  | **NOX** |  |  |
| --- | --- | --- | --- | --- | --- |
|  |  |  | Percentage |  |  |
|  | Conc | T1 | T2 | T3 | mean |
| AC-AgNPs | 25 µg/mL | 4.24 | 4.31 | 4.29 | 04.28 |
|  | 50 µg/mL | 14.58 | 14.79 | 14.71 | 14.70 |
|  | 100 µg/mL | 25.05 | 25.06 | 25.64 | 25.25 |
|  | Conc | T1 | T2 | T3 | mean |
| BHT | 25 µg/mL | 24.48 | 31.11 | 25.14 | 26.91 |
|  | 50 µg/mL | 63.98 | 60.36 | 59.71 | 61.35 |
|  | 100 µg/mL | 83.71 | 74.98 | 73.52 | 77.40 |

1. **Cytotoxicity data**

Cytotoxicity image


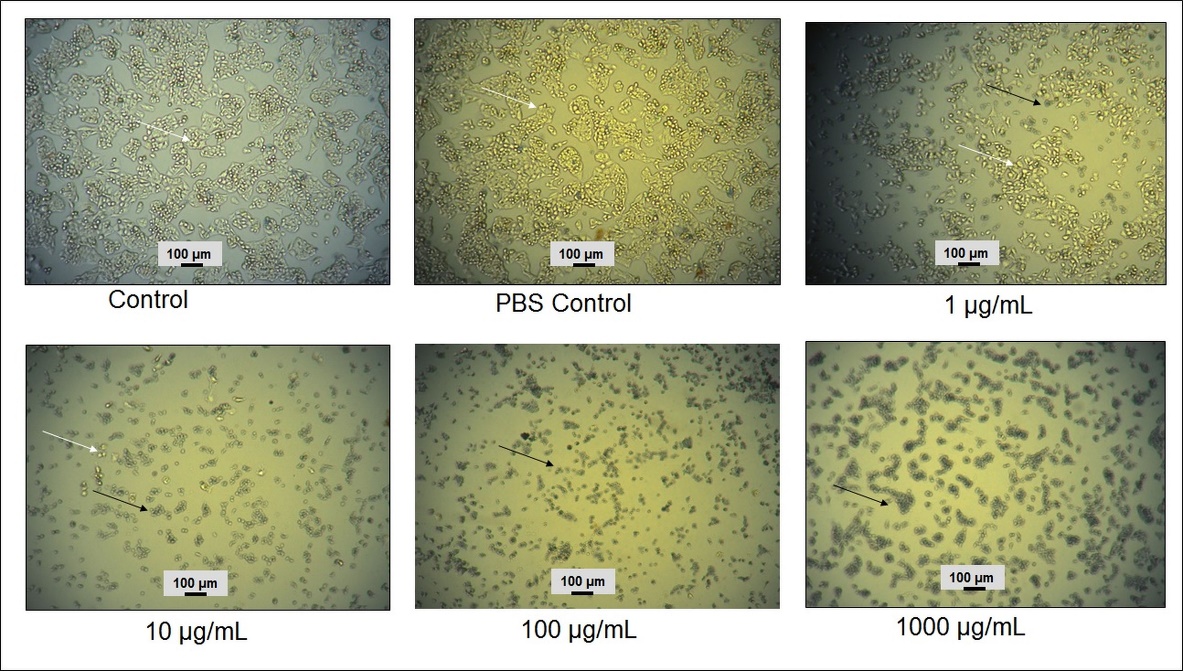


Viability percentage


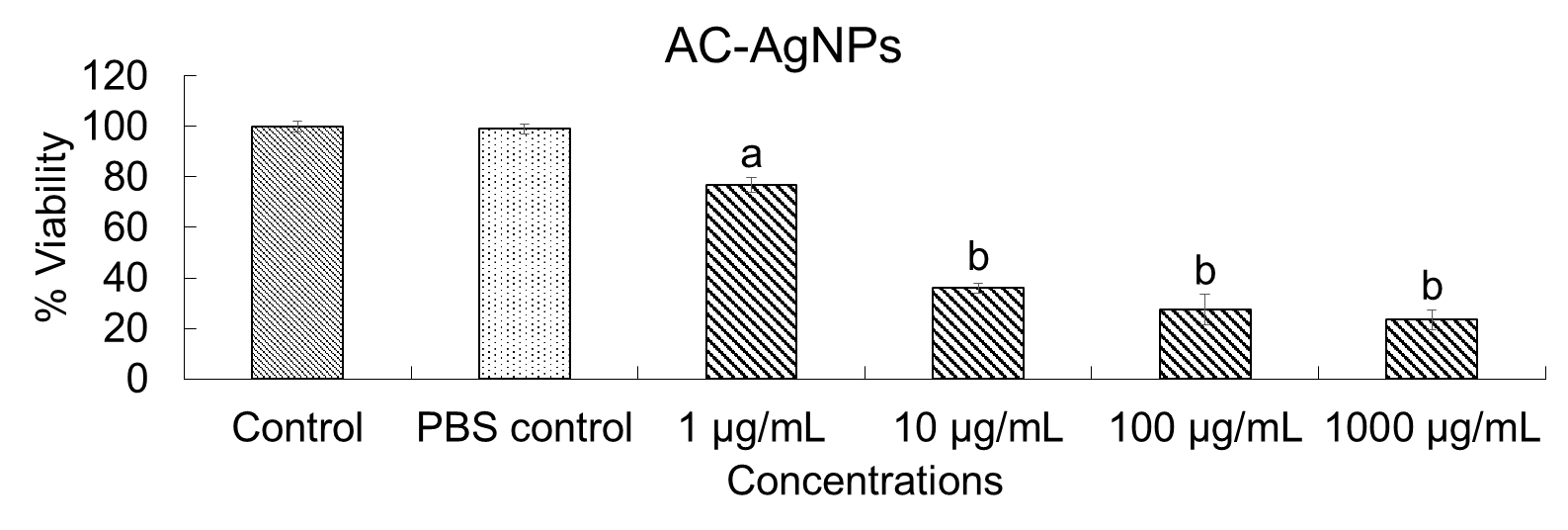


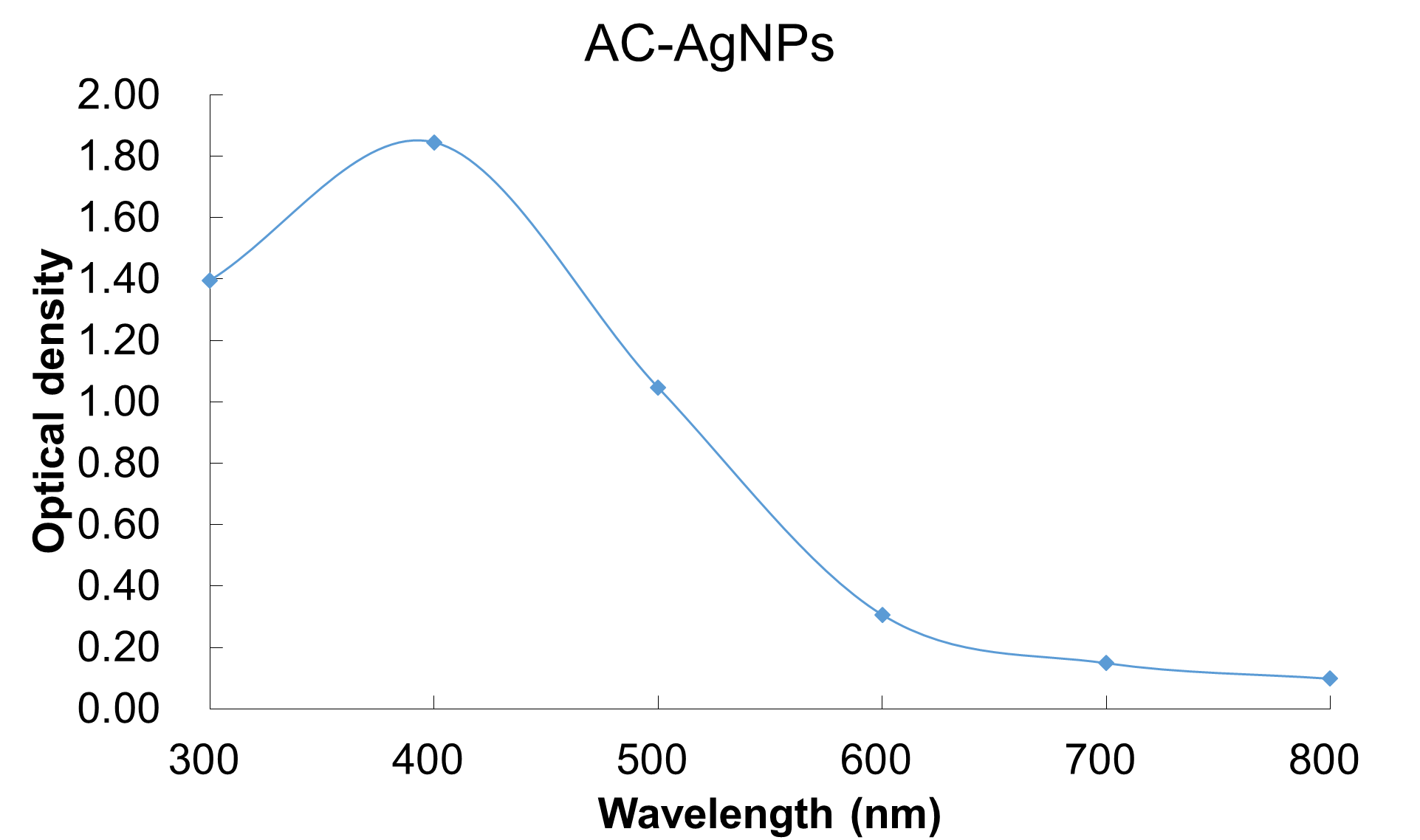


Supplementary Fig 1: Optical density of AgNPs suspended in DMEM before the treatment to HepG2 cell.

1. Alpha glucosidase assay raw data

|  | 0.008 µg/mL | 0.016 µg/mL | 0.031 µg/mL | 0.063 µg/mL | 0.125 µg/mL | 0.250 µg/mL | 0.500 µg/mL | 1.000 µg/mL |
| --- | --- | --- | --- | --- | --- | --- | --- | --- |
| Exp-1 | -0.68966 | 3.384122 | 74.03368 | 97.8348 | 98.07538 | 97.80273 | 97.7065 | 97.56215 |
| Exp-2 | 2.083772 | 20.522 | 59.94527 | 96.29552 | 97.0743 | 97.22164 | 97.20744 | 97.6122 |
| Exp-3 | -0.53879 | 11.2931 | 37.62931 | 94.13793 | 96.96121 | 97.17672 | 97.76897 | 97.93966 |
| Average | 0.285108 | 11.73307 | 57.20276 | 96.08942 | 97.3703 | 97.40036 | 97.56097 | 97.70467 |
